# The Electrophysiological Effects of Qiliqiangxin on Cardiac Ventricular Myocytes of Rats

**DOI:** 10.1155/2013/213976

**Published:** 2013-10-22

**Authors:** Yidong Wei, Xiaoyu Liu, Haidong Wei, Lei Hou, Wenliang Che, Erlinda The, Gang Li, Muktanand Vikash Jhummon, Wanlin Wei

**Affiliations:** ^1^Department of Cardiology, Shanghai Tenth People's Hospital of Tongji University, 301 Yanchang Road, Shanghai 200072, China; ^2^Department of Traditional Chinese Medicine, Shanghai Tenth People's Hospital of Tongji University, 301 Yanchang Road, Shanghai 200072, China; ^3^Zhengzhou Second People's Hospital, 90 Hanghai Road, Zhengzhou, Henan 450015, China; ^4^Department of Cardiology, General Hospital of Beijing Military Command, No. 5 Nanmencang, Dongsishitiao, Dongcheng District, Beijing 100007, China

## Abstract

Qiliqiangxin, a Chinese herb, represents the affection in Ca channel function of cardiac myocytes. It is unknown whether Qiliqiangxin has an effect on Na current and K current because the pharmacological actions of this herb's compound are very complex. We investigated the rational usage of Qiliqiangxin on cardiac ventricular myocytes of rats. Ventricular myocytes were exposed acutely to 1, 10, and 50 mg/L Qiliqiangxin, and whole cell patch-clamp technique was used to study the acute effects of Qiliqiangxin on Sodium current (*I*_Na_), outward currents delayed rectifier outward K^+^ current (*I*_K_), slowly activating delayed rectifier outward K^+^ current (*I*_Ks_), transient outward K^+^ current (*I*_to_), and inward rectifier K^+^ current (*I*_K1_). Qiliqiangxin can decrease *I*_Na_ by 28.53% ± 5.98%, and its IC_50_ was 9.2 mg/L. 10 and 50 mg/L Qiliqiangxin decreased by 37.2% ± 6.4% and 55.9% ± 5.5% summit current density of *I*_to_. 10 and 50 mg/L Qiliqiangxin decreased *I*_Ks_ by 15.51% ± 4.03% and 21.6% ± 5.6%. Qiliqiangxin represented a multifaceted pharmacological profile. The effects of Qiliqiangxin on Na and K currents of ventricular myocytes were more profitable in antiarrhythmic therapy in the clinic. We concluded that the relative efficacy of Qiliqiangxin was another choice for the existing antiarrhythmic therapy.

## 1. Introduction 

The traditional Chinese herbs have been proven safe and efficient in the management of some diseases including arrhymia since ancient times. Presently, there are four main classes of antiarrhythmic agents in clinical therapy. Three classes of them act on ion channels. As for the Chinese herb—Qiliqiangxin—few know the mechanism of antiarrhythmia. How does it impact on ion channels? Qiliqiangxin capsule is the developed Chinese herbs, which includes over 11 ingredients such as Ginseng, Radix Astragali, Aconite Root, *Salvia miltiorrhiza*, and Semen Lepidii Apetali. It was proved to be effective and safe by phase 3 clinic trial for the treatment of patients with chronic heart failure [1, 2]. Qiliqiangxin capsule also can improve heart function and decrease serum level of TNF-**α** and relieve inflammatory cell infiltration of myocardium in rats with adriamycin induced cardiomyopathy [3]. In our previous study, Qiliqiangxin affected L-type Ca^2+^ channel and blocked I_Ca-L_, as well as affected cardiac function finally. Qiliqiangxin has diphasic action that is either class IV antiarrhythmic agent or the agent for treating chronic heart failure [4, 5]. It is unknown whether Qiliqiangxin has an effect on Na current and K current because the pharmacological actions of this herb's compound are very complex. In this research, we compared the acute effects of Qiliqiangxin on the inward currents *I*_Na_ and outward currents *I*_K_, *I*_Ks_, *I*_to_, and *I*_K1_ of ventricular myocytes and hoped to demonstrate the rational usage of Qiliqiangxin.

## 2. Material and Methods 

### 2.1. Vegetal Material

Qiliqiangxin consists of Ginseng, Radix Astragali, Aconite Root, *Salvia miltiorrhiza*, Semen Lepidii Apetali, Cortex Periplocae Sepii Radicis, Rhizoma Alismatis, *Carthamus tinctorius*, Polygonatum Odorati, Seasoned Orange Peel, and Ramulus Cinnamomi [3] (Yiling Pharmaceutical Corporation, Shijiazhuang, China). The drug powder dissolved with sterile water at the concentration of 2.67 g/mL. 1 mg/L, 10 mg/L, and 50 mg/L Qiliqiangxin were prepared for the study.

### 2.2. Study Models

A total of 26 healthy Sprague-Dawley rats (9–11 weeks old, either sex, weight 210 to 300 g) were used in the study. All the rats used in the following experiments were subject to the Guiding Principles for the Care and Use of Laboratory Animals and the Recommendations from the Declaration of Tongji University. Cardiac ventricular myocytes were isolated from the hearts of rats using previous protocols [6]. Briefly, hearts were rapidly excised and cycloperfused with low calcium Tyrode's solution containing 0.08% collagenase, 0.006% protease, and then we get a single ventricular myocyte. The single ventricular myocyte selected for study is rod shaped and had clear striations and smooth and glossy surface.

### 2.3. Whole Cell Patch Clamp

 We recorded that Na current in the external solution contained (mmol/L) Choline*-*Cl 120, NaCl 25, CsCl 4, CaCl_2_ 1.8, CoCl_2_ 2, MgCl_2_ 1, HEPES 10, and Glucose 10; pH was adjusted to 7.4 with CsOH. The pipette solution contained (mmol/L) CsCl 140, NaCl 10, HEPES 5, EGTA 5, and Na_2_ATP 5; pH was adjusted to 7.3 with CsOH. *I*_Na_ was elicited from a potential −90 mV to +40 mV with 10 mV increments and by 200 ms pulses. For *I*_K_ recording, the external solution contained (mmol/L) Choline-Cl 145, MgCl_2_ 2, EGTA 1, HEPES 5, and Glucose 5.5; pH was adjusted to 7.4 with LiOH. The pipette solution contained (mmol/L) KCl 140, MgCl_2_ 1, K_2_ATP 5, HEPES 5, and EGTA 10; pH was adjusted to 7.3 with KOH. For *I*_K1_ recording, the external solution contained (mmol/L) Choline-Cl 145, KCl 5, MgCl_2_ 1, EGTA 5, HEPES 10, and Glucose 10; pH was adjusted to 7.4 with KOH. The pipette solution contained (mmol/L) KCl 140, CaCl_2_ 1, EGTA 10, HEPES 5, and K_2_ATP 5; pH was adjusted to 7.3 with KOH. For *I*_to_ recording, the external solution contained (mmol/L) NaCL 140, KCL 4, CaCL_2_ 1.5, MgCL_2_ 1, CdCL_2_ 0.5, and HEPES 5, Glucose 10; pH was adjusted to 7.4 with NaOH. The pipette solution contained (mmol/L) KCL 140, MgCL_2_ 1, K_2_ATP 5, EGTA 5, and HEPES 10; pH was adjusted to 7.3 with KOH. All recordings are at room temperature. The external solution was filled with 95% O_2_ and 5% CO_2_. *I*_K_ was elicited from the holding potential of −50 mV to +60 mV and by 4500 ms pulses with 10 mV increments. *I*_K1_ was elicited from the holding potential of −120 mV to +60 mV and by 200 ms pulses with 10 mV increments. *I*_to_ was elicited from the holding potential of −80 mV and by 300 ms pulses with 10 mV increments from a potential −40 mV to +70 mV. The effects of Qiliqiangxin: we perfuse cell with Qiliqiangxin of 1, 10, 50 mg/L, and every cell was perfused 2-3 concentration steps. To normalize for differences in total membrane area, current densities (in pA/pF) were calculated by dividing the total current by the membrane capacitance of the cell. Data were sampled at 10 kHz and filtered at 2 kHz by using an Axopatch 200 A amplifier (Axon Instruments).

### 2.4. Statistical Analysis

pCLAMP 9.0 software was used for data acquisition, and analysis values are presented as means ± S.D. Statistical comparisons between the different amiodarone concentrations groups were obtained by ANOVA. Comparisons between control and hypertrophied myocytes group means were performed with Student's *t-*test. Differences with *P* < 0.05 were considered significant, completed by SPSS 11.5 Statistically package. Concentration response relationships were fit to the Hill equation to determine the concentration of drug required for 50% inhibition (IC50).

## 3. Result

### 3.1. Current Density

The inward currents density of *I*_Na_ in cardiac ventricular myocytes (pA/pF, *n* = 10) was −56.46 ± 4.88. The outward current densities of *I*_K_, *I*_Ks_, *I*_to_, and *I*_K1_ (pA/pF, *n* = 8) were 7.27 ± 0.95, 5.68 ± 0.56, 34.71 ± 2.83, −22.82 ± 5.34 (*I*_K1_ inward), and 10.16 ± 0.20 (*I*_K1_ outward).

### 3.2. Effects of Qiliqiangxin on *I*_Na_, *I*_to_, *I*_K1_, and  *I*_K_

**Figure 1:**
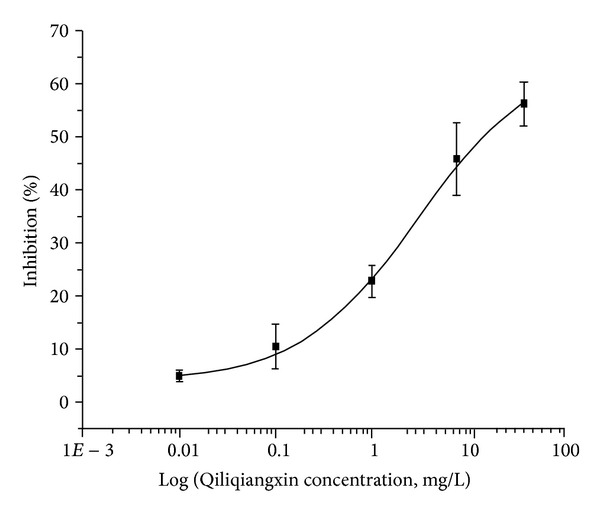
Qiliqiangxin's effect on *I*_Na_ of ventricular myocytes. Its IC_50_ was 9.2 mg/L.

**Figure 2:**
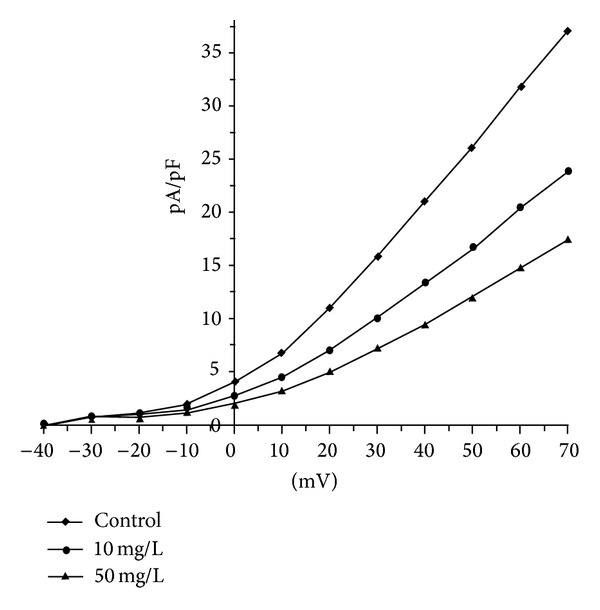
The effects of 10 and 50 mg/L Qiliqiangxin on current density-voltage curve of *I*_to_ in ventricular myocytes.

1 Mg/L Qiliqiangxin can decrease *I*_Na_ of ventricular myocytes by 28.53% ± 5.98%, and its IC_50_ was 9.2 mg/L (Figure 1). Hypoconcentration (1 mg/L) Qiliqiangxin had no effects on *I*_to_ on ventricular myocytes, while hyperconcentration Qiliqiangxin had inhibition action. 10 and 50 mg/L Qiliqiangxin decreased by 37.2% ± 6.4% and 55.9% ± 5.5% summit current density of *I*_to_ (Figure 2). For *I*_K1_, the difference concentration of Qiliqiangxin had no effects on *I*_K1_. 

**Figure 3:**
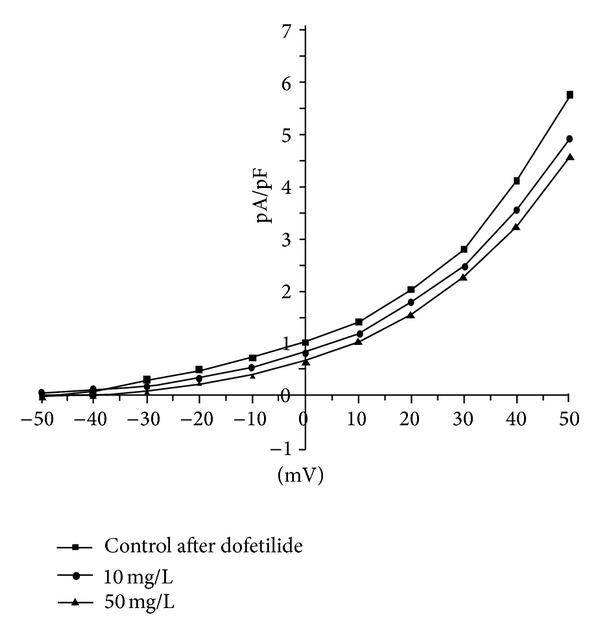
The effect of 10 and 50 mg/L Qiliqiangxin on current density-voltage curve of *I*_Ks_ in ventricular myocytes.

Delayed rectifier K^+^ current (*I*_K_) is the major outward current responsible for ventricular repolarization. 1, 10, and 50 mg/L Qiliqiangxin decreased the current density of *I*_K_ by 20.98% ± 3.97%, 31.18% ± 5.1%, and 61.52% ± 5.97%. *I*_K_ comprises two distinct current components slowly activating delayed rectifier outward K^+^ current (*I*_Ks_) and rapidly activating delayed rectifier outward K^+^ current (*I*_Kr_). We used dofetilide, a class III antiarrhythmic agent and a selective blocker of *I*_Kr_, to inhibit *I*_Kr_ and then *I*_Ks_ was recorded alone. 10 and 50 mg/L Qiliqiangxin decreased by 15.51% ± 4.03% and 21.6% ± 5.6% current density of *I*_Ks_ on ventricular myocytes (Figure 3). 

## 4. Discussion

From our previous study, Qiliqiangxin has diphasic action that is either class IV antiarrhythmic agent or the agent for treating chronic heart failure [5]. Qiliqiangxin includes over 11 ingredients. The mechanism of the antiarrhythmic action is complex and not completely understood. It should be contacted with Radix Astragal, Aconite Root, and Shensongyangxin. Radix Astragali effectively protected against cardiac dysfunctional [7]. Aconite Root was proved to have positive inotropic and chronotropic action c [8]. Shensongyangxin capsule was reported to effectively blocke *I*_Ca-L_ [9]. There were four main classes of antiarrhythmic agents in Vaughan Williams' classification. Qiliqiangxin affected L-type Ca^2+^ channel and blocked *I*_Ca-L_. It should be a class IV agent. Does Qiliqiangxin have an effect on other currents, such as Na current and K currents, because of its antiarrhythmic action? We had introduced Qiliqiangxin to test Na current and K currents. Interestingly, 1 mg/L Qiliqiangxin can decrease *I*_Na_ of ventricular myocytes by 28.53% ± 5.98%, and its IC_50_ was 9.2 mg/L. K currents are outward currents that repolarize the ventricular myocyte are numerous and complex and there is substantial interspecies variation in the profile of repolarizing currents. Prominent Ito currents have been recorded in ventricular myocytes isolated from the hearts of many species, including mice, rats, rabbits, cows, cats, dogs, ferrets and humans. In ventricular myocytes of rat, 10 and 50 mg/L Qiliqiangxin decreased by 37.2% ± 6.4% and 55.9% ± 5.5% summit current density of *I*_to_. 1, 10, and 50 mg/L Qiliqiangxin decreased current density of *I*_K_ by 20.98% ± 3.97%, 31.18% ± 5.1% and 61.52% ± 5.97%. 10 and 50 mg/L Qiliqiangxin decreased by 15.51% ± 4.03% and 21.6% ± 5.6% current density of *I*_Ks_. Acute Qiliqiangxin application can inhibit *I*_to_ and *I*_K_. 10 and 50 mg/L Qiliqiangxin had little effects on *I*_K1_, and the resting membrane potential was not affected by it. Qiliqiangxin represented class I and IV antiarrhythmic agents. It can prolong QT interval, but it does not have a similar deleterious effect as pure class III compounds. Therefore, Qiliqiangxin represented a multifaceted pharmacological profile.

 During the last decade, antiarrhythmic strategies have changed dramatically. In general, class I agents have the potential to increase mortality in patients with significant structural heart disease even though they may suppress cardiac arrhythmias. Class III and IV agents did not decrease the outcomes in clinic obviously. Qiliqiangxin is as one developed Chinese herbs, which expressed the inhibition of *I*_Na_, *I*_Ca-L_, and multi K^+^ channels. The effects of Qiliqiangxin on ventricular myocytes were more profit in clinic therapy. It is the most promising drug because of the relative efficacy and safety. Even though the mechanism of the antiarrhythmic action of Qiliqiangxin is complex and not completely understood, Qiliqiangxin is another choice for antiarrhythmic therapy at least. As for the further action in clinic of this antiarrhythmic agent, we need more research in the future.
